# Sensitizing the cytotoxic action of Docetaxel induced by Pentoxifylline in a PC3 prostate cancer cell line

**DOI:** 10.1186/s12894-021-00807-6

**Published:** 2021-03-12

**Authors:** Martha E. Cancino-Marentes, Georgina Hernández-Flores, Pablo Cesar Ortiz-Lazareno, María Martha Villaseñor-García, Eduardo Orozco-Alonso, Erick Sierra-Díaz, Raúl Antonio Solís-Martínez, Claudia Carolina Cruz-Gálvez, Alejandro Bravo-Cuellar

**Affiliations:** 1 Doctorado en Farmacología, Centro Universitario de Ciencias de la Salud, Universidad de Guadalajara, Guadalajara, Jalisco México; 2 División de Inmunología, Centro de Investigación Biomédica de Occidente del IMSS, Sierra Mojada 800, Col. Independencia, CP 44340 Guadalajara, Jalisco México; 3 Servicio de Urología, Hospital de Especialidades, CMNO-IMSS, Guadalajara, Jalisco México; 4 Centro Universitario de los Altos, Universidad de Guadalajara, Tepatitlán de Morelos, Jalisco México

**Keywords:** Pentoxifylline, Docetaxel, Prostate cancer, Apoptosis, Senescence

## Abstract

**Background:**

Prostate cancer is one of the most frequently diagnosed types of cancers worldwide. In its initial period, the tumor is hormone-sensitive, but in advanced states, it evolves into a metastatic castration-resistant tumor. In this state, chemotherapy with taxanes such as Docetaxel (DTX) comprises the first line of treatment. However, the response is poor due to chemoresistance and toxicity. On the other hand, Pentoxifylline (PTX) is an unspecific inhibitor of phosphodiesterases; experimental, and clinically it has been described as sensitizing tumor cells to chemotherapy, increasing apoptosis and decreasing senescence. We study whether the PTX sensitizes prostate cancer cells to DTX for greater effectiveness.

**Methods:**

PC3 human prostate cancer cells were treated in vitro at different doses and times with PTX, DTX, or their combination. Viability was determined by the WST-1 assay by spectrophotometry, cell cycle progression, apoptosis, generic caspase activation and senescence by flow cytometry, DNA fragmentation and caspases-3, -8, and -9 activity by ELISA.

**Results:**

We found that PTX in PC3 human prostate cancer cells induces significant apoptosis per se and increases that generated by DTX, while at the same time it reduces the senescence caused by the chemotherapy and increases caspases-3,-8, and -9 activity in PTX + DTX-treated cells. Both treatments blocked the PC3 cell in the G1 phase.

**Conclusions:**

Our results show that PTX sensitizes prostate tumor cells to apoptosis induced by DTX. Taken together, the results support the concept of chemotherapy with rational molecular bases.

**Supplementary Information:**

The online version contains supplementary material available at 10.1186/s12894-021-00807-6.

## Background

Prostate cancer (PCa) is the second most common cancer worldwide in males, and an estimated 1.28 million new cases and 358,989 deaths were reported in 2018 [1]. This cancer is among the main leading causes of cancer deaths in developed countries, with 278,539 deaths registered in 2018; however, in the United States, 31,620 deaths were estimated for 2019 [1, 2]. PCa is a clinically heterogeneous disease during which some patients may have an aggressive state of the disease with high progression and metastasis. In contrast, others have a low rate of disease progression [3]. This disease is essentially a cancer of older men. It is characterized by patterns of abnormal glandular growth in which poorly differentiated tumors are observed with a high mortality rate, while well-differentiated tumors have a favorable clinical outcome [4–7]. During the initial period of PCa, tumor growth is androgen-dependent; therefore, surgical castration and or Androgen-Deprivation Therapy (ADT) is the mainstay of treatment in metastatic Hormone-Sensitive Prostate Cancer (mHSPC) [8, 9]. Frequently the use of such treatments results in a temporary regression of the disease; however, after a time comprising 2–3 years, the tumor progresses despite continuous hormonal manipulation. This type of cancer is known as metastatic Castration-Resistant Prostate Cancer (mCRPC), [10, 11]. Cytotoxic chemotherapy remains the only treatment option in mCRPC, providing modest survival and palliative benefits [12, 13]. Taxanes represent the most active chemotherapeutic drugs that prolong survival in mHSPC and that are used as standard first-line chemotherapy and mCRPC, as second-line chemotherapy [13, 14]. Docetaxel (DTX) is one of the most important taxanes for the treatment of PCa [15]. It has been employed as treatment for 15 years, on occasion, in addition to another chemotherapeutic drug. Nonetheless, mostly as the most important treatment. DTX acts at the level of the centrosome in the mitotic spindle, thus preventing cell division [16]. However, not all patients respond to treatment with DTX due to its toxicity and a heterogenous taxane resistance, which is related to multidrug-resistant genes, *TMPRSS2–ERG* fusion genes, kinesins, cytokines, to the components of other signalling pathways [11], and a recently discovered factor: the overexpression of microRNA-323 [10, 14]. Thus, efforts have been made to improve the effectiveness of such therapeutic schemes. For its part, Pentoxifylline (PTX) (1-[5-oxohexyl]-3, 7-dimethylxanthine) is a synthetic derivative of the methylxanthines, initially developed as a hemorheological agent for circulatory problems and considered as a non-selective adenosine antagonist [17]. Currently, its clinical usefulness is due to its anti-inflammatory, antioxidant, and immunomodulatory properties [18, 19]. The anti-inflammatory action of PTX lies in its blocking of proinflammatory cytokine production (IL-1, IL-6, and TNF-α) by increasing cyclic Adenosine Monophosphate c (cAMP) limiting the formation of ATP [20]. In previous studies, a decreased activity in the Transcription Factor kB (NF-κB) was described as an antitumoral manner, through the inhibition of IκB phosphorylation [21].

In this respect, our work group previously found, clinically and experimentally, the sensitization to PTX-mediated chemotherapy of different drugs, such as Adriamycin (ADM), Cisplatin, and Perillyl Alcohol in cervical cancer cells and in other tumors, increasing apoptosis and decreasing cell senescence [22–26]. There are also reports of sensitization to radiotherapy in prostate cancer lines in which PTX induces a cell cycle arrest in the G2 phase [27]. The aim of the present study was to determine the effects of PTX in combination with DTX in an in vitro model with the PC3 cell line from a castrate-resistant prostate cancer.

## Materials and methods

### Drugs

Docetaxel (DTX) was obtained from Pisa Farmacéutica, México, stored at 4 °C for fewer than 4 days, and adjusted to the desired concentration with F-12K culture medium immediately before use. Pentoxifylline (PTX), (Sigma, St. Louis, MO, USA) was dissolved in sterile saline solution at a concentration of 0.5 M and maintained at − 4 °C for fewer than 4 days.

### Cell line

We worked with the PC3 cell line (ATCC CRL 1435; Manassas, VA, USA). This cell line is epithelial, derived from bone metastasis of an independent androgen grade-IV prostate adenocarcinoma of a 62-year-old caucasian patient and was authenticated using the Multiplex Cell Authentication system by Multiplexion GmbH (Friedrichshafen, Germany) report 2386 and tested for mycoplasma contamination using the Universal Mycoplasma Detection Kit (ATCC, Manassas, VA, USA), and the cells were negative throughout the study.

### Cell culture

**Fig. 1:**
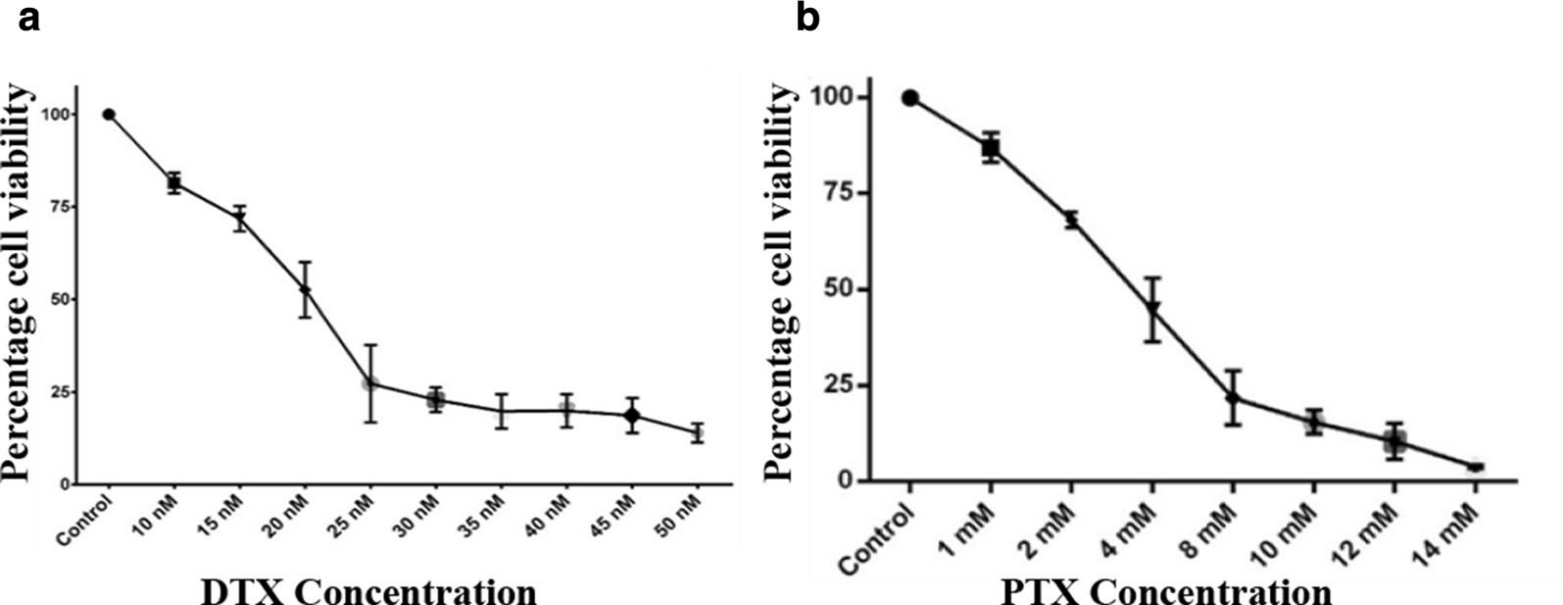
DTX and PTX dose–response curve. PC3 prostate cancer cells (3 × 10^4^) were incubated and exposed to different concentrations of DTX (**a**) or PTX (**b**). Viability was evaluated by the WST-1 assay. The results represent the mean ± SD of the normalized percentages of the three independent experiments carried out in triplicate

PC3 cells (1 × 10^6^ cells) were cultured at 37 °C in an atmosphere containing 5% CO_2_ and 95% air, in 150-cm^2^ culture flasks for adherent cells (Corning CLS430825) suspended in 18 mL of F-12K Medium (Kaighn’s Modification of Ham’s F-12 Medium) from GIBCO (Invitrogen Co.) with the addition of 10% Bovine Fetal Serum (BFS) (GIBCO), 1% 100X L-glutamine solution (GIBCO), and antibiotics/antimycotics (Penicillin–Streptomycin-Neomycin). This supplemented culture medium will be designated F-12KS and was replaced every 48 h. Prior to the experiments, PC-3 cells were detached with Accutase (GIBCO™) [28]; then, the cells were washed 3 times in PBS 4 °C, pH 7.4, and live cells were then resuspended at the desired concentration in F-12KS culture medium. Depending on the experiment, live PC3 cells determined by Trypan Blue exclusion (> 95%) were seeded at concentrations of 1 × 10^4^ to 1 × 10^6^ cells in 6-, 48-, or 96-well plates and cultured for 24 h before the application of treatments. The ideal concentrations of the treatments were determined by means of a dose–response curve and kinetics at 24, 48, and 72 h (Fig. 1).

### In vitro treatments

The groups utilized were as follows: an Untreated Control Group (UCG) as negative control; an 8 mM PTX group; a 25 nM DTX group, and a group for 8 mM PTX + 25 nM DTX. In all experiments, PC3 cells treated with PTX were administered 1 h before DTX, in that there is evidence that better results can be obtained in this manner [21, 22]. In the same way peripheral blood monocluear cells from healt voluntary were obtained and treated with PTX (4 and 8 mM) during 24 h to investigate toxicity of PTX in normal cells.

### Cell viability assay

The effects of both drugs on the cells were determined by the WST-1 4-(3-(4-Iodophenyl)2-(4-nitrophenyl)2H-5-tetrazolium)-1,3-benzene disulfonate) assay. This study is based on the reduction of tetrazolium salts into formazan; the rate of WST-1 cleavage by mitochondrial dehydrogenases correlates with the number of viable cells. Exponentially grown cells were harvested and seeded in 96-well plates (1 × 10^4^ cells/well) and allowed to attach to wells overnight. After 24 h, the medium was replaced with fresh medium and then the cells were treated according to the treatment scheduled at 24 h, 48 h and 72 h. After the incubation, 10 μL/well of WST-1/ECS reagent (Quick Cell Proliferation Colorimetric Assay Kit WST-1; BioVision, Inc. Milpitas, CA, USA) was added to each well and the PC3 cells were incubated for another 2 h. Absorbance was measured in a microtiter plate reader (Synergy™ HT Multi-Mode Microplate Reader; Biotek, Winooski, VT, USA) at 450 nm. The percentage of viability was calculated utilizing the following formula: viability = (1 − [absorbance of experimental well − absorbance of blank)/(absorbance of untreated control well − absorbance of blank) × 100%. Data are reported as the percentage of cell viability in comparison to that of its respective UCG considered as 100% [29]. The IC_50_ of the DXT or PTX treatment in PC3 cells was determined from survival curves generated for each experiment. Data are reported in percentage ± Standard Deviation (SD) of cell survival as compared with the UCG considered as 100% [30].

### Assessment of apoptosis

Apoptosis was evaluated by flow cytometry after treatment with 8 mM PTX, 25 nM DTX, and PTX (8 mM) + DTX (25 nM) employing the Annexin-V-Fluos staining kit (Roche, Basel, Switzerland) according to the manufacturer’s instructions. For this, 1 × 10^6^ PC3 live cells from each of the different experimental conditions were treated. Etoposide at a concentration of 1 μg/mL was used as positive control and UCG as a negative control. The cells were harvested using Accutase as described previously and were washed 3 times with PBS. After that, the cells were then resuspended in 200 µL of incubation buffer, which contained 3 μL of Annexin V-Fluorescein Isothiocyanate (FITC) and 5 µL Propidium Iodide (PI) (1 µg/mL stock solution). These were mixed gently and incubated at 20 °C for 10 min in the dark. Finally, 400 μL of incubation buffer was added to the suspension, which was analyzed by flow cytometry. At least 20,000 events were acquired with Attune (Applied Biosystem), and the analysis was performed using. FlowJo ver. 7.6.5 software (Tree Star, Inc., Ashland, OR, USA). Annexin V-FITC-negative and PI-negative cells were considered live cells. Cells positive for Annexin V-FITC but negative for PI were regarded as being in early apoptosis. Cells positive for both Annexin V-FITC and PI were taken as undergoing late apoptosis. Cells positive for PI and negative for Annexin V-FITC were considered necrotic. The data will be represented as the mean ± SD of the percentage of cells and represent the addition of early and late apoptosis.

### ELISA apoptosis assay

For the determination of the apoptosis assay by histone-associated fragmented DNA, 2 × 10^4^ PC3 live cells were seeded per well (200 μL volume) in a 96-well plate and were treated under the same conditions described above for 24 h at 37 °C, 95% air, and 5% CO_2_. The cell plates were centrifuged at 1200 rpm for 10 min at 4 °C. Supernatants were decanted and the cells were lysed for 30 min in 200 μL of lysis buffer, centrifuged at 200 × *g* for 10 min. Twenty μL of lysate of each sample was transferred onto the Streptavidin-coated microplate plus 80 μL immunoreagent per well. The samples were incubated for 30 min and were protected from light at between 15 and 25 °C. The cells were centrifuged at 1200 rpm for 10 min at 4 °C, and 20 μL of the supernatant from each well was taken and placed into the ELISA 96-well kit plate. Eighty μL of immunoreactive was added to each well (Incubation Buffer 72 μl, Anti-Histone 4 μl, Anti-DNA 4 µL), the plates were covered with an adhesive cover, and these were incubated in a shaker at 300 rpm for 2 h at between 15 and 25 °C. The plates with the supernatants were removed. Each plate well was washed 3 times with 300 µL of incubation buffer; 100 μL of ABTS solution was added to each well. The plates were incubated in a shaker at 250 rpm for 20 min; 100 μL of ABTS stop solution was added to each well. The absorbance of each sample was determined using a microplate reader (Sinregy HT Multi-Mode Microplate  Reader, Biotek at 450 nm). In the DNA fragmentation test, the rate of apoptosis is reflected by the Enhanced factor (fold change) of mono- and oligonucleosomes accumulated in the cytoplasm, both of which were calculated and normalized versus UCG.

### Caspases activity assay by flow cytometry

The generic caspase activity (Caspases-1, -3, -4, -5, -6, -7, -8, and -9) was determined by flow cytometry using the Generic Caspase Activity Assay kit (Abcam, Cambridge, UK). The activation of caspase is an indicator for cell apoptosis, TF2-VAD-FMK is a fluorescent reporter that binds to active caspases in apoptotic cells. Briefly, PC3 cells were seeded at a density of 1 × 10^6^ cells in 6-well plates and treated for 24 h with the different drugs as described previously. After this, the cells were harvested and collected by centrifugation, suspended in 500 μL of F-12KS. Then, 1 μL of 500X TF2-VAD-FMK was added and incubated at room temperature for 1 h. Finally, the cells were washed with PBS at 4 °C pH 7.4 and resuspended in 500 μL of assay buffer for immediate determination. The samples were processed using Attune™ Applied Biosystem flow cytometry equipment. For each sample, at least 20,000 events were analyzed with the FlowJo ver. 7.6.5 software (Tree Star, Inc; Ashland, OR, USA). Data are presented as a percentage of caspase-positive cells.

### ELISA assay to determine caspases activity-3, -8, and -9

The activity of caspases-3, -8, and -9 was measured using the Caspases Colorimetric Assay kit (Abcam) following the manufacturer’s protocol. PC3 cells (5 × 10^6^) were cultured and treated for 24 h under the conditions previously described. Afterward, the cells were washed with PBS at 4 °C, pH 7.4, 50 μL of cell lysis buffer was added and incubated on ice for 10 min, homogenized was centrifuged at 10,000 × *g* for 1 min. The protein concentration was determined by Bradford assay (Bio-Rad, CA, USA), and 100 μg of protein was used for each test. Subsequently, the absorbance at 405 nm was determined in a microplate reader (Synergy HT Multi-Mode Microplate Reader Biotek). Results are represented as a percentage of caspase activity and compared with the respective percentage in UCG cells, considered as 100%.

### β-galactosidase-associated senescence

Cell senescence was evaluated by flow cytometry using the C12FDG kit (5-Dodecanoylaminofluorescein Di-β-D-Galactopyranoside; Invitrogen), which acts as a substrate for β-galactosidase. Briefly, PC3 cells were seeded at a density of 1 × 10^6^ cells in 6-well plates and treated for 24 h with the different drugs. Afterward, we added 100 nM of bafilomycin A1 for 1 h at 37 °C afterwards the cells were washed, harvested, collected by centrifugation, and incubated with 10 μM C12FDG (Invitrogen) according to the manufacturer’s instructions. Acquisition of the samples was carried out in Attune (Life Technologies, Carlsbad, CA, USA) flow cytometry equipment. For each sample, at least 20,000 events were analyzed with Attune cytometer and the data were analyzed with the FlowJo ver7.6.5.  Software (Tree Star, Inc). Data are expressed by the percentage of positive senescent cells compared with the respective percentage in UCG cells (considered as 100%). In Additional file 1: Figure S1 is shown the gating strategy for apoptosis, generic caspase activity, and senescence analyzed by flow cytometry.

### Determination of the cell cycle by flow cytometry

For the cell-cycle analysis, the PC3 cells were initially synchronized. In brief, cells were cultured in F-12K culture medium containing 5% FBS for 12 h. Subsequently, the cells were washed with PBS and were incubated in serum-free medium for 18 h. Finally, the cells were split and were released into cell cycle by the addition of 10% FBS in F-12K culture medium. A total of 1 × 10^6^ PC3 cells were treated with each drug alone or with both drugs for 24 h. The BD Cycletest Plus DNA reagent kit was utilized according to the manufacturer’s instructions (BD Biosciences, San Jose, CA, USA). DNA QC Particles (BD, Biosciences) equipment was employed to verify the instrument performance and quality control of the equipment used for cell cycle analysis (Attune cytometer; Applied Biosystems). The samples were analyzed by flow cytometry (Attune; Life Technologies, Carlsbad, CA, USA). For each sample, at least 20,000 events were analyzed with the Attune Cytometric Software PC v2.1(Life Technologies). The percentage of cells represents the cell cycle distribution in the G1, S, and G2 phase; this was assessed by using the obtained data and processed with FlowJo ver. 7.6.5 software (Tree Star, Inc., Ashland, OR, USA) [31].

### Statistical analysis

Each experiment was carried out in triplicate and repeated on at least three occasions. The results are expressed as the mean ± Standard Deviation (SD) of the values obtained. The difference between groups was determined by the non-parametric Mann‒Whitney *U* test considering a significant difference of *p* < 0.05. In some experiments, the Δ% was calculated, which represents the percentage of increase or decrease in relation to UCG.

## Results

### Determination of IC_50_ of PTX and DTX in PC3 cells of prostate cancer

The IC_50_ of PTX and DTX were determined in PC3 cell cultures treated for 24 h. After receiving the results, we observed that both drugs produced a decrease in the viability effect in a dose-dependent manner. The IC_50_ is reached at a concentration of 20 nM DTX (Fig. 1a). Thus, a concentration of 4 mM of PTX was sufficient to reach IC_50_ (Fig. 1b). However, we were also able to observe the antitumor activity of this drug, which decreases 25% of the cell viability at a concentration of 2 mM.

### Effect of PTX alone or in combination with DTX on the cell viability of PC3 cells

The viability of PC3 cells incubated with different doses of PTX + DTX, using concentrations close to the IC_50_, was determined by WST-1 assay. Table 1 shows the results of the percentage of viability achieved after the administration of treatments at 24, 48, and 72 h. It presents an evident decrease in viability by combining DTX and PTX, compared versus the UCG. We also observed similar results with both doses of PTX 4 and 8 mM (*p* < 0.05). With this kinetic and dose–response evaluation, we concluded that the ideal concentrations of treatment for our future experiments will be DTX at 25 nM and PTX at 8 mM, alone or combined, for each respective experimental group. Surprisingly, also in Table 1, it can be observed that PTX, mainly at 4 and 8 mM, by itself reaches levels of cytotoxicity comparable to those obtained with the combination of both drugs in particular to 48 and 72 h. In contrast to prostate tumor cells, the viability of normal peripheral blood mononuclear cells practically was not afected by the PTX (3 ± 0.9 and 5 ± 1.3% decreases in viability for the doses of 4 and 8 mM respectively).

**Table 1:** Viability of PC3 cells treated with PTX alone or in combination with DTX

GROUP/HOURS	PTX			DTX		PTX + DTX	
	2 mM	4 mM	8 mM	15 nM	25 nM	4 mM/25 nM	8 mM/25 nM
24	70.0 ± 3.6	34.7 ± 8.2^**+**^	26.3 ± 2.6^+^	87.3 ± 2.1	45.8 ± 2.5	24.7 ± 2.5^+^	22.3 ± 3.2^+^
48	61.7 ± 4.2	29.4 ± 3.3^+^	19.7 ± 3.1^+^	69.5 ± 2.1	42.3 ± 7.4	23.7 ± 2.5^+^	21.3 ± 4.2^+^
72	59.1 ± 2.2	20.7 ± 1.5^+^	17.3 ± 3.5^+^	62.3 ± 3.2	25.7 ± 2.1	19.2 ± 2.3^+^	17.3 ± 1.5^+^

PTX decreases viability, alone or in combination with DTX, in PC3 cells after 24, 48, and 72 h of treatment. A total of 3 × 10^4^ PC3 cells were seeded and were initially treated (24 h prior) with different concentrations of PTX and DTX, alone or combined. Later, cell viability was evaluated by WST-1 assays. The results represent the mean ± SD of the values obtained of three experiments carried out in triplicate. Untreated control cells were considered as 100%. + Mann‒Whitney *U* test, *p* < 0.05 versus DTX-treated groups

### Effect of PTX in apoptosis induction by DTX in the PC3 line cell

**Fig. 2:**
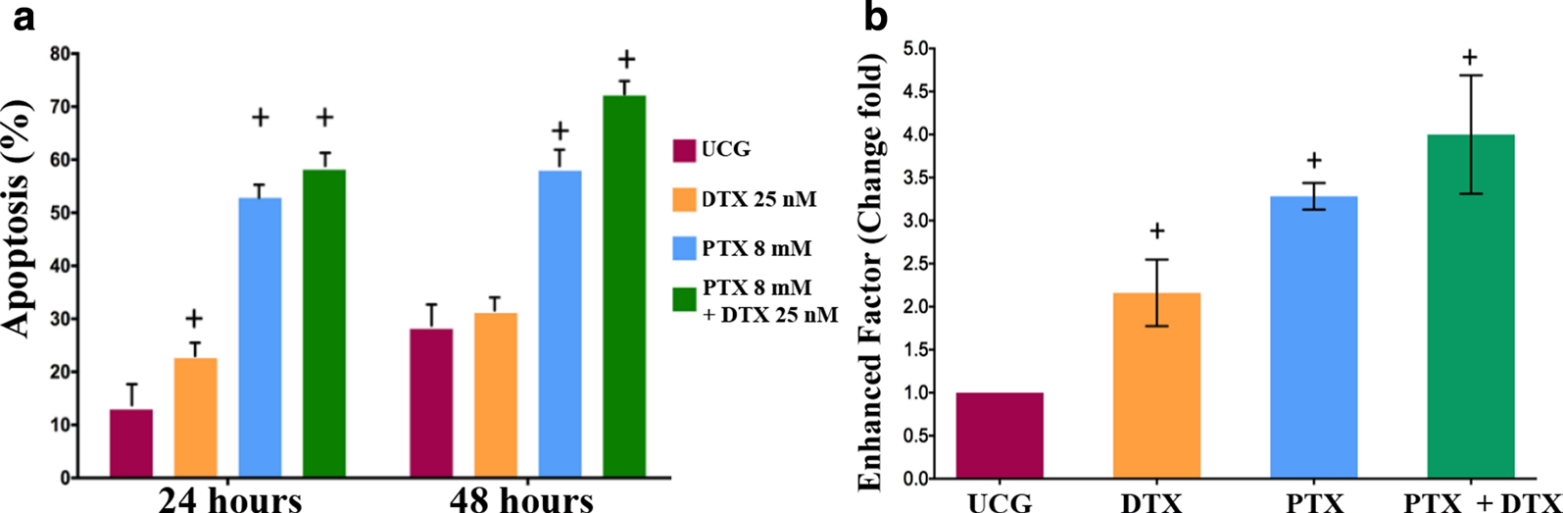
Apoptosis of PC3 cells treated with DTX, PTX, or their combination. The 1 × 10^6^ live cells were seeded and exposed to DTX 25 nM, PTX 8 mM, or PTX 8 mM + DTX 25 nM. **a** Apoptosis was evaluated by Flow Cytometry using Annexin V/PI assay. **b** Apoptosis was evaluated by ELISA, the results were normalized in relation to UCG, and represent the mean ± SD of three independent assays. Statistical analysis in both Figures: Mann‒Whitney *U* test, *p* < 0.05 in all PTX-treated groups versus UCG and DTX groups

Initially, apoptosis induction was studied by Annexin V/Propidium Iodide (IP) assay (Fig. 2a). After 24 h of treatment, we found a statistical difference between treated groups compared versus the UCG; however, it is noteworthy that the percentage of apoptosis was considerably higher in the groups treated with PTX + DTX or with PTX even in comparison with the DTX group (*p* < 0.005). At 48 h, we found the same behaviour with higher values: the PTX and PTX + DTX groups exhibited apoptosis of 57.6% and 72.1%, respectively, compared with 30% of apoptosis in DTX and UCG (*p* < 0.005). Furthermore, apoptosis was determined by apoptotic DNA fragmentation using a sandwich ELISA assay. Figure 2b depicts the results, noting a significant increase of apoptosis in the PTX + DTX group (Δ% = 298% above that of the UCG). Similarly, the DTX (Δ% = 115%) and PTX (Δ% = 225%) groups also demonstrated increased apoptotic activity compared with the UCG group (*p* < 0.05 all PTX-treated groups vs. UCG and DTX groups). Taken together, the results concluded with the sensibilization in DTX by PTX, increasing apoptosis in PC3 tumor cells. Thus, PTX reveals antitumor activity per se, a novel effect of this drug.

### PTX alone or in combination with DTX induces caspase activity in PC3 cells

**Fig. 3:**
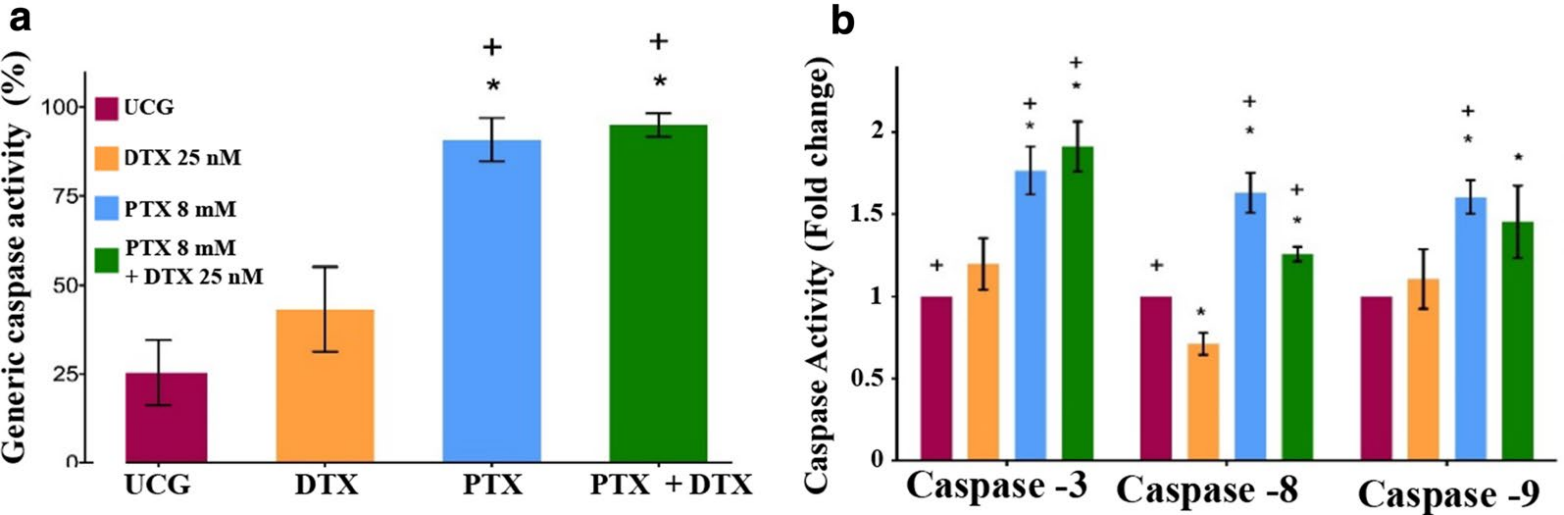
Caspase activation in PC3 tumor cells treated with PTX, DTX or their combination. The 2 × 10^5^ PC3 cells were cultured and exposed to DTX 25 nM, PTX 8 mM, or PTX 8 mM + DTX 25 nM for 24 h. **a** General caspase activity was determined by flow cytometry. **b** Caspases-3, -8, and -9 activity was determined by sandwich ELISA assay. The results were normalized in relation to UCG, and represent the mean ± SD of the three experiments carried out in triplicate. Statistical analysis: Mann‒Whitney *U* test *(*p* < 0.05) versus UCG, + (*p* < 0.05) versus DTX 25 nM

The role of caspases in apoptosis was analyzed. First, we determined the general caspase activity (Fig. 3a), and afterward, we studied in particular caspase-8 (extrinsic pathway), caspase-9 (intrinsic pathway), and caspase-3 (common pathway) activity (Fig. 3b). The treated groups produced greater general caspase activity at a level that was statistically significant compared to that of the UCG group (*p* < 0.05) being higher and comparable between themselves in PTX and PTX + DTX-treated groups (*p* < 0.005 vs., the DTX group). However, we can observe that DTX does not receive sufficient caspase activation in the intrinsic and extrinsic pathways to find statistically significant results compared with the UCG (*p* > 0.05). On the other hand, the PTX group demonstrated a preference for caspase-9 activation (*p* < 0.05 vs., all groups), PTX + DTX also achieves higher labels of caspase-9 activation, near those found in the PTX group (*p* < 0.05 vs. DTX and UCG). Meanwhile, in PTX-treated groups, both pathways are activated, finding a notable increase of caspase-8 activation that is statistically significant as compared with the UCG and DTX groups (*p* < 0.05). The PTX + DTX group exhibited a similar activation of caspase-3 to that of the PTX group, but higher than the caspase-3 activation in the UCG- and DTX-treated groups (*p* < 0.05), with these results, taken together, indicating that PTX plays an important role in the induction of apoptosis.

### Role of PTX in the induction of the senescence of PC3 cells treated with DTX

**Fig. 4:**
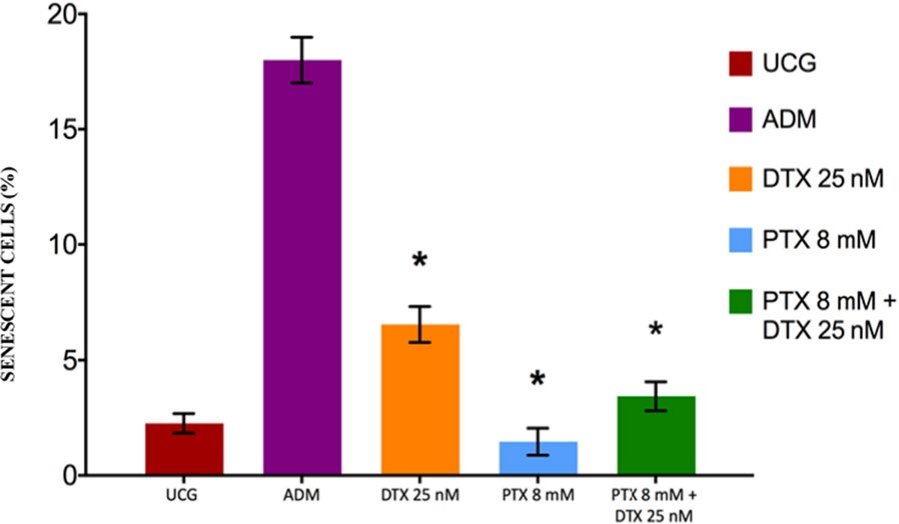
Senescent PC3 cells treated with DTX, PTX or their combination. The 1 × 10^6^ PC3 cells were cultured and treated with DTX 25 nM, PTX 8 mM, or PTX 8 mM + DTX 25 nM for 48 h. Cell senescence was determined by the levels of the β-galactosidase enzyme by flow cytometry. UCG = Untreated Control Group, ADM = Adriamycin (ADM) use as a positive control. The results represent the mean ± SD of the three replicated experiments. Statistical analysis: Mann‒Whitney *U* test *(*p* < 0.05) versus UCG, + (*p* < 0.05) versus DTX 25 nM

The levels of the β-galactosidase enzyme were determined to measure senescence. Built on the premise that chemotherapy induces senescence, there is major logical senescence in the DTX group, as reported in Fig. 4, which shows a significant increase compared with the control group (Δ% = 192%). For its part, the senescence values of the PTX group demonstrated lower values than UCG (Δ% = − 35%), but are statistically and significantly lower compared with the DTX group, since it represents a Δ% = − 77% (*p* < 0.05). The PTX + DTX group exhibited a smaller percentage of senescence than DTX, but slightly higher than the PTX-treated group.

### Cell cycle phases arrested in PC3 cells treated with PTX and/or DTX

The main objective of this experiment was to determine how PTX + DTX could modify the cell cycle in all experimental groups. In Additional file 2: Figure S2 is shown a representative example of cell cycle analysis using FlowJo software.

**Fig. 5:**
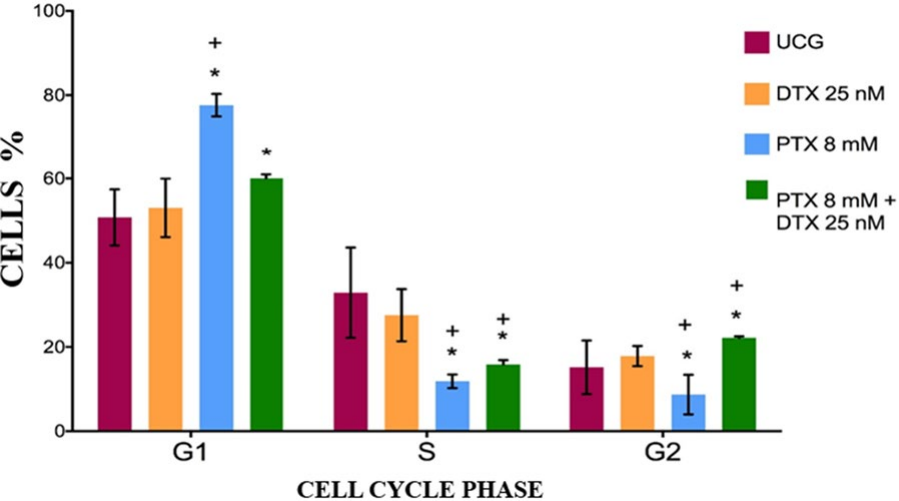
Cell cycle phases arrested in PC3 cells treated with PTX and/or DTX. The 1 × 10^6^ PC3 cells were cultured and treated with DTX 25 nM, PTX 8 mM, or PTX 8 mM + DTX 25 nM for 24 h. The cell cycle was determined by flow cytometry. The results represent the mean ± SD of the three replicated experiments. Statistical analysis: Mann‒Whitney *U* test *(*p* < 0.05) versus UCG, + (*p* < 0.05) versus DTX 25 nM

Flow cytometry analyzed this through the DNA obtained from PC3 cells after 24 h of treatment. Figure 5 shows that, during in the G1 phase, we found that the higher percentage of cells corresponds to the group treated with PTX (77.7% ± 2.7%), followed by the PTX + DTX group (60% ± 1.0%), both with a significant difference (*p* < 0.05) compared with the UCG group (50.8% ± 6.7%) and the DTX group (53.0 ± 7.8%), which is strictly comparable with UCG. Furthermore, PTX alone or combined with DTX showed no arresting activity during the S phase and activity lower than the close percentages of the UCG and DTX groups (UCG- and DTX vs. PTX-treated groups *p* < 0.05). Finally, the lower percentage of cells in phase G2 corresponding to the group of PC3 cells treated exclusively with PTX 8.7 ± 2.1% (*p* < 0.05 vs. the other groups), and the other groups revealed very close percentages to about 20%. On the other hand, we found an arresting cell cycle during the G2 phase by PTX + DTX, a statistically significant increase compared with the control and the DTX group (*p* < 0.05), but with values very close to those the other groups.

## Discussion

In the present work, it is shown that PTX can sensitize PC3 prostate cancer cells to the toxicity of the DTX, and it is essential to note that the PTX also demonstrates antitumor activity per se. The importance of the latter is that metastatic prostate cancer has a poor prognosis, and DTX is the first line of treatment for this tumor [8, 15].

Both drugs, either isolated or in combination, show a clear dose- and time-dependent effect on the survival of the PC3 tumor cells, indicating the treatments’ specificity-of-action. The doses of PTX are in agreement with previous clinical and experimental observations with other tumor cells, such as leukemias, lymphoma, cervical cancer, and retinoblastoma [22, 23, 29, 32, 33].

On the other hand, it is well known that the most critical effect of chemotherapy against tumor cells is the induction of apoptosis [34]. In the present work, we observed that PTX could sensitize prostate tumor cells to the toxic effects of DTX, increasing apoptosis significantly in terms of the apoptosis induced by DTX.

In addition, with regard to this point, it is noteworthy that the label of antitumor activity exhibited by PTX per se is near those showed revealed in the PTX + DTX-treated cells; this is also in agreement with previous observations with respect to other tumor cell lines and supports the idea of the possible utilization of PTX as a direct antitumor drug [23], but additional studies are necessary to confirm this possibility.

The fact that strictly similar observations have been reported with ADM, Cisplatin, and Perillyl Alcohol permits us to think that PTX have a common target in different tumor cells with the same effects, sensitizing tumor cells to chemotherapy and showing per se an antitumor effect [22, 23].

The apoptosis observed in the PTX-treated groups, as depicted in Fig. 3, is dependent on caspases involving both extrinsic and intrinsic apoptotic pathways. The most important of these is the mitochondrial pathway, and the higher labels of caspase activity principally in the intrinsic pathway in PTX-treated groups can explain the persistence of the most top labels of apoptosis observed and afford security to our observations. In addition, the differences observed with caspases suggest a specific mechanism of action of each treatment, the main effect of DTX is die the cells and PTX have other effects, this may explain the difference of caspase activity between different groups.

The senescence initially was considered as a defence mechanism of the cells against the malignization of the cells. However, this is now in doubt, because cells in senescence are live and secrete factors that favor a tumor microenvironment that facilitates their growth and expansion and an inhibitor of the immune response [28]. In the present work, we observed that PTX does not induce senescence and that it diminishes the senescence produced by DTX. Therefore, the use of PTX is an advantage because a defence mechanism of the tumor cells against chemotherapy is the induction of senescence, as we can observe in the DTX group (Fig. 5) and is inhibited by the PTX.

We observed, in all groups, that the higher percentage of cells were in the G1 phase; however, this was more intensely observed in PTX and PTX + DTX groups. Concerning phase G2, lower results were observed with the PTX and PTX + DTX groups and, as expected, the lower percentages found in phase S was practically without a difference between groups. The importance of these observations is that it is well-known that, in the G0 and G1 phase cycles, tumor cells are more sensitive to the toxic effects of chemotherapy [35, 36]. Additionally, the finding that PTX-treated groups also showed a lower percentage of these in phase S, strongly suggesting the lower ability of PC3 cells to divide.

It is important to note that there was an agreement with all of the experiments carried out, in that the work helps to support the use of PTX in Oncology. It also has been reported that increased chemotherapy efficiency [18, 23, 32, 37] inhibits side effects of chemo- and radiotherapy [18], which is certain even when used in children with leukemia, and in addition exerted an antitumor impact per se [22, 23]. Another possible advantage of PTX pretreatment is that as the tumour cells are more sensitive to chemotherapy, so requiring lower doses of chemotherapy avoiding or reducing side effects, at this respect it was observed experimentally in lymphoma-bearing mice treated with PTX + ADM survived more than 1 year after receiving only one half of the standard therapeutically active ADM dose compared to single treatment of ADM [29].

In conclusion, PTX sensitizes prostate PC3 cells to DTX toxicity increasing apoptosis. The agreement of the results of this work, as well as the previous reports in the literature, provide additional evidence supporting the concept of chemotherapy with rational molecular bases [29].

## Supplementary Information

**Additional file 1.** **Figure S1**: Representative analyses of apoptosis, generic caspase activity and senescence in PC3 cells treated or not with PTX, DTX or PTX + DTX.

**Additional file 2.** **Figure S2**: Representative cell cycle analyses of PC3 cells treated or not with PTX, DTX or PTX + DTX.

## Data Availability

The datasets used and/or analysed during the current study are available from the corresponding author on reasonable request.

## Abbreviations

cAMP: Cyclic adenosine monophosphate c
ADT: Androgen-deprivation therapy
DTX: Docetaxel
mHSPC: metastatic Hormone-sensitive prostate cancer
PTX: Pentoxifylline
PCa: Prostate cancer
mCRPC: metastatic Castration resistant prostate cancer
ADM: Adryamicin
UCG: Untreated control group
