# Supplementary material for: Sensitizing the cytotoxic action of Docetaxel induced by Pentoxifylline in a PC3 prostate cancer cell line
Source: BMC Urol. 2021 Mar 12;21:38. doi: 10.1186/s12894-021-00807-6 (PMC7953714; doi:10.1186/s12894-021-00807-6)
Supplement: Supplementary file 1 — Additional file 1. Figure S1: Representative analyses of apoptosis, generic caspase activity and senescence in PC3 cells treated or not with PTX, DTX or PTX + DTX. [file 12894_2021_807_MOESM1_ESM.pptx]

## Slide 1
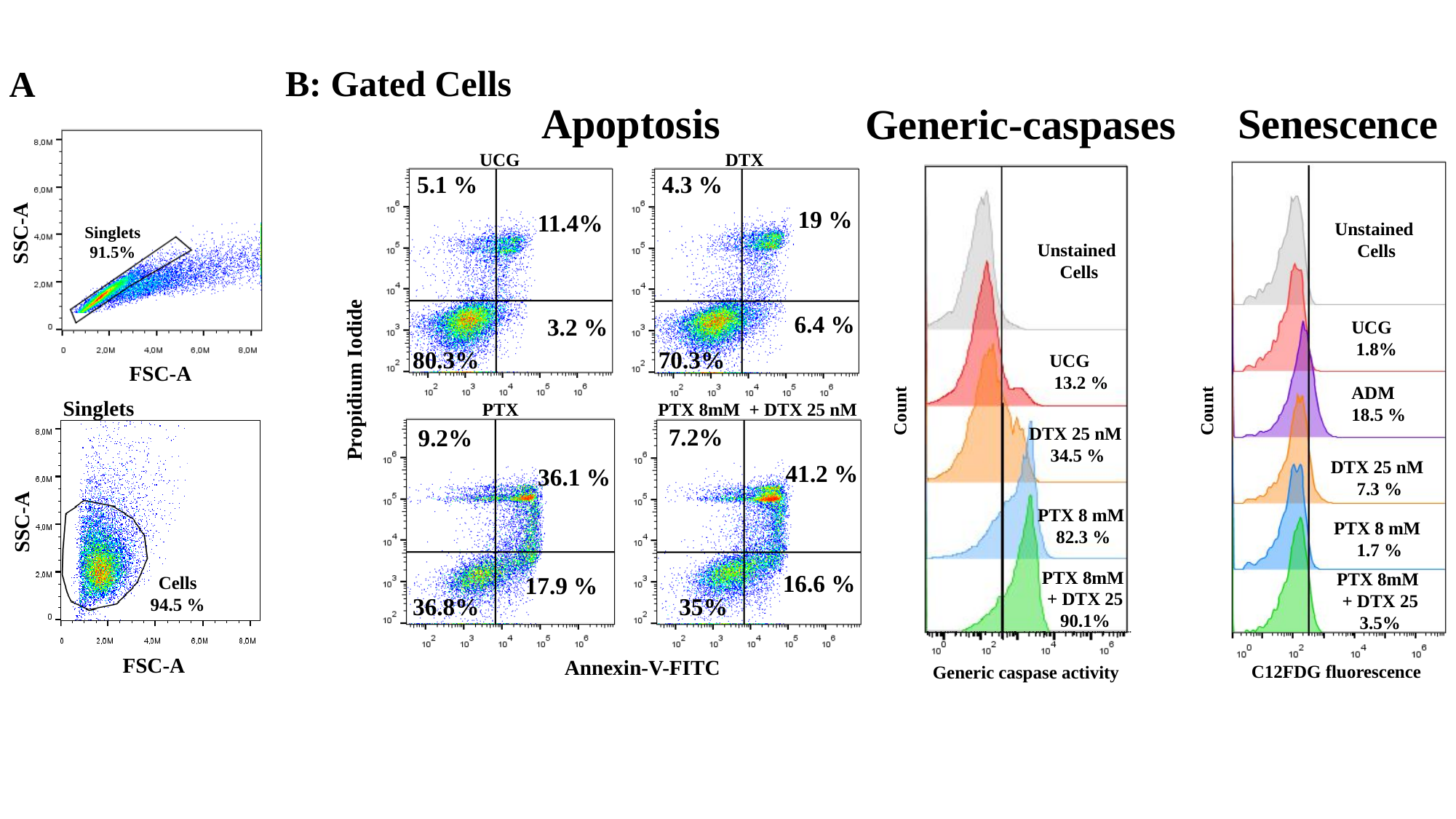

B: Gated Cells
A
Senescence
Apoptosis
Generic-caspases
DTX
UCG
 5.1 %
4.3 %
19 %
11.4%
Unstained
 Cells
SSC-A
Singlets
91.5%
Unstained
 Cells
6.4 %
3.2 %
UCG
 1.8%
70.3%
80.3%
UCG
 13.2 %
FSC-A
Propidium Iodide
ADM
18.5 %
Singlets
PTX
PTX 8mM + DTX 25 nM
Count
Count
DTX 25 nM
 34.5 %
7.2%
9.2%
DTX 25 nM
 7.3 %
41.2 %
36.1 %
PTX 8 mM
 82.3 %
SSC-A
PTX 8 mM
 1.7 %
PTX 8mM
+ DTX 25
90.1%
16.6 %
PTX 8mM
+ DTX 25
3.5%
17.9 %
Cells
94.5 %
35%
36.8%
FSC-A
Annexin-V-FITC
C12FDG fluorescence
Generic caspase activity
