# Supplementary figures and images for: Sensitizing the cytotoxic action of Docetaxel induced by Pentoxifylline in a PC3 prostate cancer cell line
Source: BMC Urol. 2021 Mar 12;21:38. doi: 10.1186/s12894-021-00807-6 (PMC7953714; doi:10.1186/s12894-021-00807-6)

## Slide 1
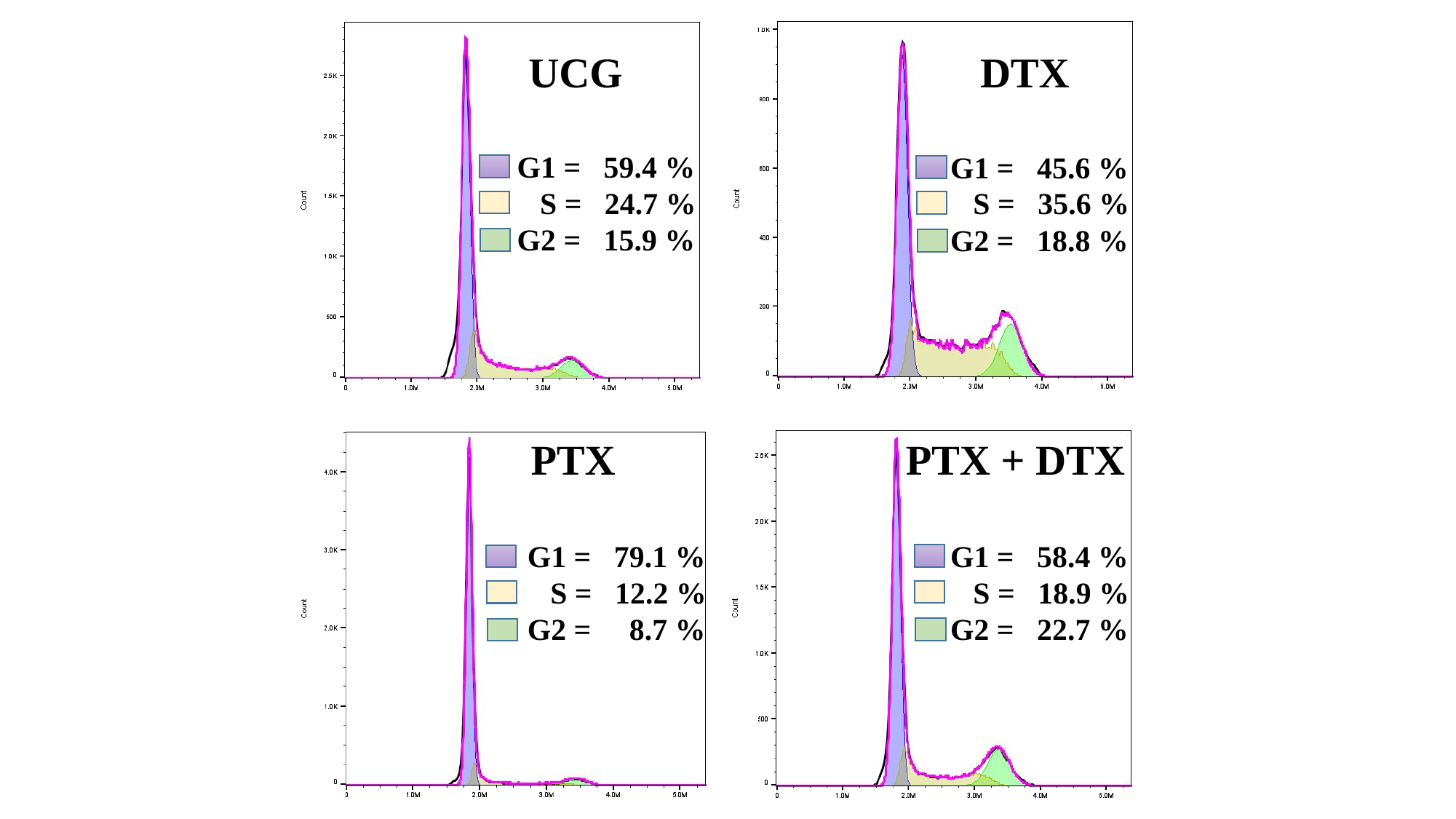

UCG
DTX
G1 = 59.4 %
 S = 24.7 %
G2 = 15.9 %
G1 = 45.6 %
 S = 35.6 %
G2 = 18.8 %
PTX
PTX + DTX
G1 = 79.1 %
 S = 12.2 %
G2 = 8.7 %
G1 = 58.4 %
 S = 18.9 %
G2 = 22.7 %

Supplement: Supplementary file 2 — Additional file 2. Figure S2: Representative cell cycle analyses of PC3 cells treated or not with PTX, DTX or PTX + DTX. [file 12894_2021_807_MOESM2_ESM.pptx]
